# The role of community leaders on adolescent’s HIV and sexual reproductive health and rights in Mulanje, Malawi

**DOI:** 10.1186/s12978-020-00917-8

**Published:** 2020-05-14

**Authors:** Chancy S. Chimatiro, Precious Hajison, Adamson S. Muula

**Affiliations:** 1 School of Public Health and Family Medicine, College of Medicine, University of Malawi, Blantyre, Malawi; 2 Africa Center of Excellence in Public Health and Herbal Medicine (ACEPHEM), College of Medicine, University of Malawi, Blantyre, Malawi; 3 PreLuHa consult, Namiwawa Street, Newroard location, PO BOX 703, Zomba, Malawi

**Keywords:** Adolescents, Community leaders, Sexual and reproductive health

## Abstract

**Background:**

We conducted this study to understand roles of community leaders on adolescent’s HIV and Sexual and Reproductive Health (SRH) rights in Mulanje-Malawi. We discussed how each role can influence health seeking behaviour and improve SRH rights among adolescents from the local perspective.

**Methods:**

A qualitative study approach was used. We conducted 17 Key Informant Interviews (KIIs) and 12 Focus Group Discussions (FGDs) with community leaders. Purposive sampling technique was used to select study participants for KIIs. We also used purposive sampling technique to identify two villages from each of the six Traditional Authorities (TAs) where FGDs were conducted. All participants in FGDs were purposively selected. Inductive thematic content analysis was done guided by the study objectives to generate emerging themes.

**Results:**

Community leaders have many roles on adolescents HIV and SRH. These roles include advisory, encouragement, regulating and restricting cultural practices, formulating bye-laws and handling sexual abuse complaints. However, community leaders with religious affiliation have shown to have different views with those representing other institutions not affiliated to religion. In addition, the majority of community leaders indicated low level of knowledge on adolescent’s SRH rights.

**Conclusion:**

We suggest that the roles of community leaders differ depending on the position held and institution represented. Those not affiliated with religious institutions can encourage certain behaviour in adolescents while those from religious background are discouraging it. Stakeholders involved in the fight against HIV, promotion of SRH and rights should invest more on capacity building among the community leaders.

## Plain English summary

Community leaders play important roles among their subjects in communities around Malawi. They establish and maintain norms and cultural values within their societies. However, there is paucity of data concerning specific roles of community leaders on adolescent’s HIV, SRH and rights. This study was conducted inorder to find out about the roles of community leaders on adolescent’s HIV, SRH and rights and how they influence health seeking behaviour.

The study participants were asked to mention their roles as community leaders and how they influence adolescent’s health. Data collection was done through Key Informants Interviews (KIIs) and Focus Group Discussions (FGDs).

The study indicated various roles which include advisory, encouragement, regulating and restricting cultural practices, formulating bye-laws and handling sexual abuse complaints performed by the community leaders.

In conclusion, the roles of community leaders differ depending on the position held and institution represented. Therefore, this study recommends that stakeholders involved in the fight against HIV, promotion of SRH and rights should invest more on capacity building among the community leaders.

## Background

Community leaders are considered as an important element for successful community development in Malawi and some parts of Africa [1]. They play a prominent role in establishing and maintaining norms as well as decision-making at the community level [2]. In South Africa, for example, it was reported that community leaders were effective in engaging their communities that gave an opportunity for the people-centeredness decision making [3]. In Malawi, there are both established and non-established traditional leadership positions like Traditional Authorities (TAs), Group Village Heads (GVHs), Village Heads (VHs), church leaders and initiation leaders (*anakungwi or angalibas*) [4] who are considered as custodians of culture, society values and customs [5]. They are held in high esteem among their followers and can assist in shaping behaviour of adolescents [1]. They are sources of information to their subjects including on health [6]. In addition, they were reported to have assisted in reducing maternal deaths in Mchinji district, Malawi, by introducing regulation that ban home baby deliveries [7]. They are decision makers and regulate cultural practices and beliefs within their societies [8].

In another study done in Nigeria, it was reported that one of the major problems in addressing adolescents SRH was the low use of services due to social-cultural barriers and limited access to the health services [9]. This put community leaders at the centre stage as they are the custodians of cultural practices and beliefs. The adolescents in Malawi face many challenges with their SRH. About 13% of girls and 22% of boys in Malawi began having sexual intercourse before the age of 15 [10]. This has resulted into 29% of adolescent girls getting early pregnancies [10]. Moreover, young people including adolescents contribute to one-third of all new HIV infections in Malawi [11]. Adolescent pregnancies are associated with poor outcomes such as induced abortions, still births, school dropout [12] and maternal deaths [13].

The majority of people in Malawi (84%) lives in rural areas under community leaders [14]. It has been estimated that over 83.3% of girls and 84.1% of boys [15] are living in rural areas. The median age at sexual debut among adolescents in Malawi is 16.8 and some of them have more than one sexual partner. To curb the practice of early marriages among adolescents, the Malawi government emphasize on the importance of involving traditional leaders during program planning for the health interventions [16].

Furthermore, traditional leaders have been reported as one of the most important stakeholders in the fight against HIV and promotion of good health in the communities [17]. They have ability to influence their subjects through multiple routes. Mobilization and sensitisation, formation of bye-laws, use of fines, fear and coercion have been mentioned in other studies as some of the ways used by the community leaders in influencing their communities [1, 4, 17]. Although performing many roles, there is paucity of data specifically about the roles of community leaders on adolescents HIV and Sexual Reproductive Health in Malawi. It is important that the roles of community leaders be studied, this may enable policy makers to include their views during planning for adolescent health interventions and other relevant policies. This study, therefore, was conducted in Mulanje-Malawi to determine the roles of community leaders on adolescent’s HIV, SRH and rights. Mulanje is one of the district in Malawi with high adolescents HIV prevalence (7.1%) [18] and early pregnancies (31.6%) [6].

## Methods

### Study design and setting

**Fig. 1:**
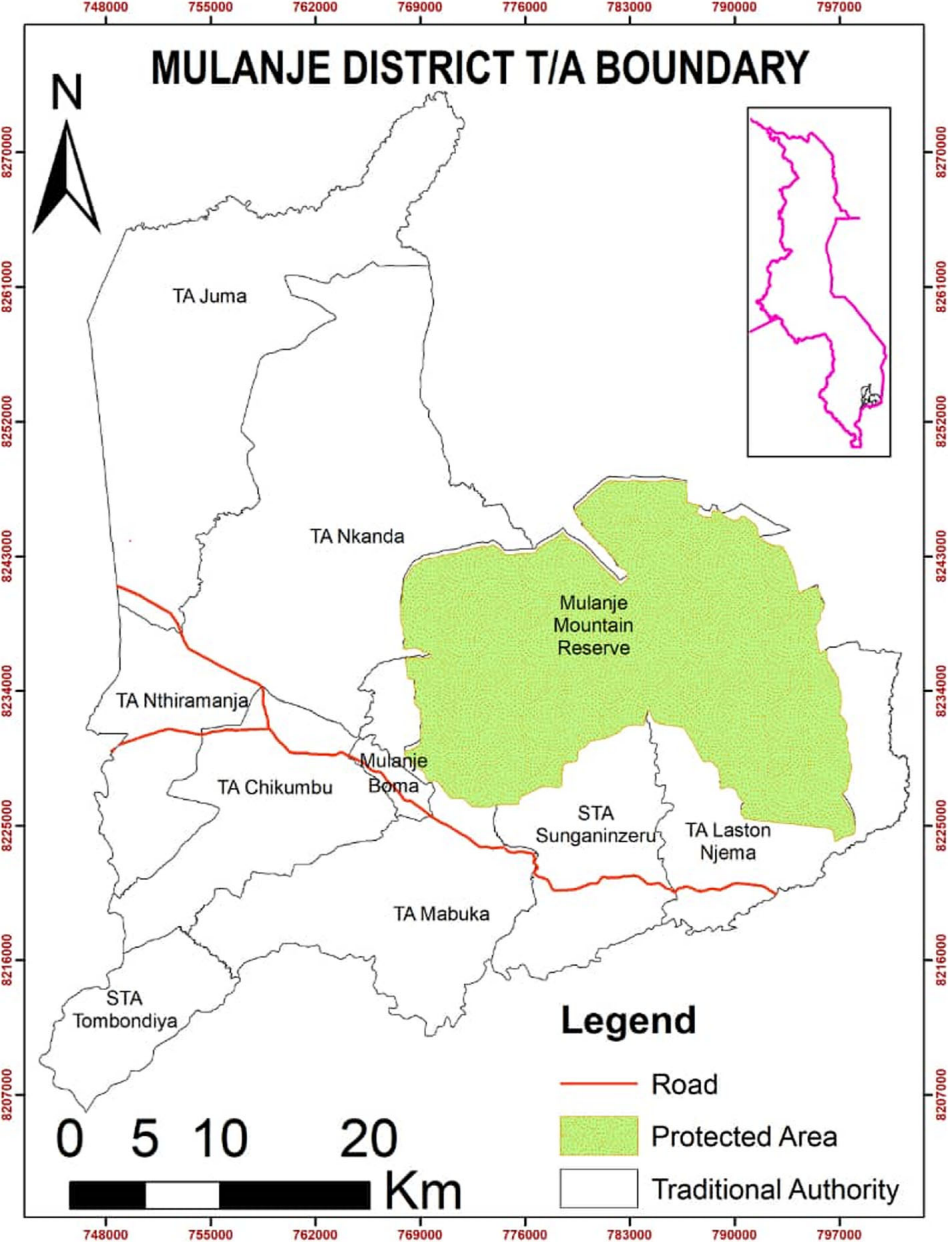
Map of Mulanje

We conducted an exploratory qualitative study in Mulanje district, Malawi (see Fig. 1). The district has 6 Traditional Authorities (TAs) namely; Mabuka, Mthiramanja, Mkanda, Chikumbu, Njema and Juma. We used two methods, Key Informant Interviews (KIIs) and Focus Group Discussions (FGDs). The two methods were used inorder to increase the credibility and validity of the results. In addition, the methods were chosen inorder to get an indepth and broader understanding of the roles of the community leaders on adolescent’s HIV and sexual reproductive health and Rights. Figure 1: Map of the study district, Mulanje, Malawi.

### Sample size and sampling technique

In total, the study had 118 participants of which 17 took part in Key Informant Interviews (KIIs). We included five Traditional Authorities (TAs), four Group Village Headmen (GVHs), four Religious leaders and four Initiation Counsellors in KIIs. We had 101 participants who took part in Focus Group Discussions (FGDs). Participants in FGDs included the Group Village Headmen (GVH), religious leaders, Family Ethnic Elders and initiation counsellors. The study participants in FGDs were of mixed ages, both males and females. We used purposive sampling technique to select KIIs participants. We purposively selected two villages from each of the six TAs where FGDs were conducted. All participants in the FGDs were also purposively selected. The sample size was reached at after saturation point whereby no more new answers were coming out from the study participants.

### Data collection

We recruited and trained two Health Surveillance Assistants (HSAs) to be part of the research field team for a period of 1 week. We recruited those community leaders who held leadership positions and resided in the area for more than 1 year. Apart from note taking, the field team used two voice recorders to ensure that the collected data is safe in case one develop mechanical faults during and after interviews. We conducted the interviews at a place preferred by the study participants. We assured our study participants that participation into the study was voluntary and they can terminate the interviews whenever they feel so.

### Data management

We created pass word accessible file where the data was kept. We listened to the proceedings of the interviews several times before transcribing the data from local language Chichewa to English. The transcriptions were read several times before data analysis for familiarisation and identification of themes emerged. We assigned each theme with appropriate code.

### Data analysis

We sorted and analysed data using inductive thematic content analysis by grouping of similar themes, ordering and structural through utilization of matrices. The emerged themes were given appropriate codes. The codes were being assigned under the main theme it supported.

We identified a language expert from the department of languages, chancellor college, University of Malawi, who transcribed the data from the local language Chichewa to English. CC and PH read each individual transcript many times over to identify similar patterns of the roles of the community leaders. CC and PH coded the data manually before exporting to Nvivo 11 for the final analysis. AM reviewed, refined the emerged themes and supervised the whole process. The themes emerged from the transcripts were advisory, encouragement, regulating and restricting cultural practices, formulating bye-laws and dealing with domestic violence. The main ideas from each study participant were isolated and assigned under the specific theme it supported.

### Ethical review and approval

The study was reviewed and approved by the College of Medicine Research and Ethics Committee (COMREC), (certificate number P.08/18/2449). Permission to conduct the study in Mulanje district was granted by the District Commissioner. The study participants were identified by numbers in order to ensure privacy and confidentiality. All study participants consented and an impartial witnesses were requested to consent for participants who could not read and write. All participants consented for the peer review and publications of the study results.

## Results

In total, we conducted 12 FGDs and 17 KIIs. The study participants described different roles that are performed by community leaders in protecting adolescents from HIV infection and improve SRH and rights. Notably, the roles included advisory, regulating cultural practices and beliefs, advocacy and protection over domestic violence.

### Advisory role on health life for adolescents

We found that the majority of community leaders advises adolescents on abstinence to avoid contracting HIV. One of the key informant said: *“I always talk with adolescents on the risk of HIV and how they can prevent it, I mostly put much emphasis on the need for them to abstain from premarital sex because in doing so they cannot contract HIV.”* (Key informant 12). Community leaders also reported that there are some adolescents who cannot resist from sexual intercourse. Such adolescents are advised to use condoms. One of the key informants reported that: “*Some of the adolescents cannot stand the temptation of having sexual intercourse due to modern technologies as most of these youth have modern cell phones they use to browse pornographic videos (****zithunzi zolaula****) as such I strongly encourage them to use condoms when they are in that situation.”* (Key informant 13).

However, some community leaders expressed different views on advising adolescents to use condoms. A participant reported that: *“I don’t discuss issues concerning condom use with adolescents because it’s like I am encouraging them to commit adultery.”* (Key informant 6). Another participant said, *“It’s against our belief to encourage adolescents to use condoms whenever they have a desire to have sexual intercourse.”* (FGD 5, respondent No 7).

The community leaders indicated that they advise adolescents on the importance of HIV testing and counseling. They reported that knowing the HIV status can help the adolescents to make decisions that could prevent the spread of HIV. One of the participant reported that, *“I advise adolescents to go and have HIV test at the health centre to ascertain their HIV status.”* (Key informant 3).

The community leaders indicated that they also encourage those living with HIV to continue taking their Anti-retroviral drugs *“It is our role to ensure that those adolescents living with the HIV are living positively and stay health, so we encourage them to continue taking their drugs daily and whenever they are not feeling well, we encourage them to seek medical attention.”* (FGD 12, respondent number 1).

### Encouragement role on safe behaviour towards prevention of HIV and SRH

The community leaders explained that they encourage adolescent boys on medical male circumcision instead of going for tradition circumcision. Traditional male circumcision is conducted by the untrained local initiation counsellors in most of the rural areas of Malawi. This was mostly disclosed by the community leaders affiliated to the religious denominations. One of the religious leaders reported that: *“Our religion demands that male children should be circumcised and in the past we used to send them to ndagala (Initiation camps) where they were being circumcised by the ngalibas (local initiation counsellors). Due to the HIV, we are now sending our children to the health facilities where they are circumcised first before enrolling them into initiation camps.”* (Key informant 13).

The majority of female community leaders reported that they have the responsibility to encourage adolescent girls to go and access cancer screening service at the health facilities when they have reached an adulthood age. A female community leader reported that: *“Whenever adolescents have gathered at one place, I always have an opportunity to discuss and encourage them to go and have cancer screening at the health facility when they are old enough as I believe in early preparation (mmera npoyamba).”* (Key informant 4). This claim was affirmed by another community leader who reported that: *“I advocate for safe motherhood issues within this area and most of the times, when I chat with adolescents, I also inform them on the dangers of cervical cancer”* (FGD 6, respondent 1).

Some of the community leaders reported to encourage adolescents on contraceptive use. This was reported by the majority of the community leaders in both FGDs and KIIs. One of the community leaders in a FGD reported that: *“I sometimes advise adolescents to consider taking contraceptives especially girls whenever they feel that they cannot convince their boyfriends to use condoms so that they can avoid early pregnancies.”* (FGD 4, respondent 6). Likewise, in KII interviews another community leader supported this by saying: *“I know there are some adolescent girls who do not take our advice seriously and don’t like the issue of condom use, so I always ask them to go to the health facilities and get contraceptives to avoid pregnancy.”* (Key informant 11).

There were also mixed feelings among the community leaders about advising adolescents on contraceptive use. Some community leaders reported that they do not advise adolescents to use contraceptives such as pills, injections, intra-uterine devices and Norplant as that might encourage them to practice unprotected sex and be exposed to STIs including HIV. A respondent said: *“I do not advise adolescents to use pills or injections because it may look like I am encouraging them to practice unsafe sexual intercourse.” (*A female respondent, FGD 10).

Several community leaders reported to encourage the adolescents on their education. However, long distances to schools was mentioned as a challenge faced by many adolescents. This was highlighted in FGDs and KIIs. In acknowledgement of this claim, one respondent in FGD said: *“As leaders we took time to encourage adolescents to concentrate on their education if they are to become reliable leaders in the future.”* (FGD 5, respondent 6). This was also reported in KIIs as another key informant said: *“Whenever I have called for a meeting with my subjects I remind them on the need to encourage adolescents on education, however, the long distance to schools and lack of hostels makes it difficult to monitor the behavior of adolescents as some take advantage of that to indulge in bad habits including sexual intercourse.”* (Key informant 8).

### The role of regulating and restricting harmful cultural practices and beliefs that fuel HIV and deterred SRH rights

The community leaders also indicated that they have a role in determining what should be practiced or not within their communities by restricting harmful cultural practices that fuel spread of HIV. The majority of the community leaders reported that they condemns harmful cultural practice of cleansing (*kusasa fumbi*). A community leader reported that: “*I personally restrict anakungwi and ngalibas (initiation councsellors) never to tell any adolescent that once out of initiation camp should have sexual intercourse to cleanse themselves. If anyone disobeys this order I do ban such person from practicing in my area apart from fining him/her.”* (A male respondent, KIIs 1). This was also highlighted in the FGDs and one of the respondents said: *“Much as we are supposed to safeguard our culture but we also have responsibility to ensure that we stop all harmful cultural practices and beliefs that increases the spread of HIV because things are changing. During our days we had curable diseases (meaning other STIs) but now we have AIDS that cannot be cured, so I always discuss with my subject to restrict some harmful cultural practices that promote spread of HIV in their respective villages.”* (FGD 2, respondent 11).

### The role of formulating and advocating for bye-laws that guide adolescent SRH

The community leaders indicated that they perform many roles on adolescent SRH in their respective areas. They perform these roles to prepare and safeguard adolescents from problems that come with early pregnancies. The community leaders reported to have a role in formulating bye-laws to guide adolescents SRH within their areas. They advocate for these bye-laws to be discussed at the district level during full council meetings. One of the key informants reported that: *“We formulate bye-laws like ensuring that no adolescent is married before reaching an adulthood age which we present during council meetings at DCs office”* (Key informant, number 16). In agreement to this, another traditional leader reported that: *“In my area, I have informed all my subjects to ensure that no adolescent fails to attend school because of early marriage or employment, if anyone found doing that can be punished by paying MK10, 000.00 and everyone is fully aware of this.”* (Key informant number 3).

### The role of dealing with sexual abuse

The community leaders also reported that they have a role in dealing with domestic violence including sexual abuse. They perform this role by either bringing the perpetrators and punish them under the local laws or reporting them to police. A female community leader reported that: *“When I receive a report that an adolescent has been sexually abused, I make sure that the culprit is brought to book and punished. Sometimes I report such cases to police if the issue is beyond us like rape cases”* (Key informant no 16). In agreement to this, another community leader reported that: *“I have been sending some issues to the police for their intervention, I remember one of the cases was to do with an adolescent who was impregnated by her step father, however, there are some issues we discuss here and if punishable under local laws then we normally do so, otherwise we don’t just refer each and every issue to police.”* (A male community leader, FGD 5).

### The role of promoting youth friendly reproductive health services (YFRHS)

Majority of the community leaders were not sure of the YFRHS available at their nearby health facilities hence they are unable to inform adolescents on that. One of the community leaders reported that; *“To be honest with you I don’t know anything about the YFRHS.”* (Key informant 2). However, some of the community leaders indicated that they are aware of the YFRHS at their health facilities. A community leader said, *“I have heard of the YFRHS at our health facility but I don’t know exactly what type of services are provided to these adolescents, so I don’t advise them anything on that.”* (FGD 6, respondent 2).

The community leaders also mentioned that they face some challenges as they perform their duties when advising adolescents. They indicated that lack of parental support, inadequate knowledge on SRH issues and adolescent’s rudeness were among some of the challenges. One of the community leaders reported that, *“Some adolescents are rude and they cannot listen to you when advising them which discourages some of us*.”

### How do the roles influence health seeking behaviour among adolescents

We further explored the influence of the roles of the community leaders on adolescent’s health seeking behaviour. The community leaders reported that the advice they give to the adolescents are giving positive outcomes. They reported that many of the adolescents are able to access health services when the need arises. One respondent in FGD reported that: “*There is positive response from the adolescents nowadays, I have seen some of them going to the health facility for HIV test unlike how things were before*.” (FGD 2, respondent 9).

The community leaders also reported an improvement on SRH as adolescents are able to access services including the contraceptives. A study participant reported that: *“I have seen some adolescents especially boys coming to me complaining about shortage of condoms at the health facility. To me this is the sign of positive results of our discussions with them, its encouragement on our part.”* (KII No 14). Another participant reported that: *“I have seen many girls getting contraceptives especially injection which is an indication that they are taking our advice seriously.”* (FGD 10, respondent 7).

### Ways of improving adolescents SRH rights from local perspective

The community leaders mentioned various ways of improving adolescents SRH rights in their communities. The ways mentioned include trainings, community awareness and use of leaflets and addition of SRH rights on education curriculum.

### Training the community leaders on SRH rights

The majority of the community leaders mentioned the need to train them on the adolescents SRH and Rights to improve their knowledge. A community leader said, *“If I can be trained on the adolescents SRH and rights then I will be able to advocate for that as I will have enough knowledge about the rights. Sometimes I fail to answer basic questions concerning the rights as a leader but if trained I can easily answer some of the questions.”(*Key informant 13). A village Head said, *“Hahaha ok I can say that let those who have information whether from government or NGOs come and train us, may be from there we will have enough information about the importance of the SRH rights*.” (FGD 10 participant 1).

### Intensifying community awareness and advocating for the SRH rights through village action groups (VAGs)

Some of the community leaders mentioned the need for intensifying community awareness. This will enable many of the community leaders to access messages and have knowledge on SRH rights. Some of the community leaders mentioned of the VAGs as vehicles for strengthening adolescent SRH and rights within their communities. A participant reported that: *“Let the NGOs take a leading role through community awareness. This will assist some of us to be conversant with adolescent SRH rights.”* (FGD 18, respondent No 11). Another participant reported that; *“In our communities we have what we call community actions groups (meaning VAGs) who are championing various projects, these people can also be used to promote adolescents SRH and rights if properly trained.”* (FGD 9, respondent 5).

### The use of printed leaflets

Some of the community leaders suggested on the use of the print out leaflets as a way of promoting adolescents SRH and rights within their communities. The printed leaflets in both languages, Chichewa and English, should be displayed in common places like community grounds, health facilities and school notice boards. One of the community leaders said; *“I can read and write and if I can get a printed leaflet concerning adolescents SRH and rights I can easily read, especially one in our local language “Chichewa.”* (A female respondent No 2, FGD 4). A female key informant said; “*Although I didn’t go very far with school but at least “a yekha ndimatomuziwa” (meaning she can manage to read some basic printed materials in local chichewa language) but I don’t have chance of having these print-outs about adolescents SRH and rights.”* (Key informant 2).

### Inclusion of the SRH rights into education curriculum

Some of the community leaders were of the opinion that SRH and rights should be included in the education curriculum. They suggested that this will assist the adolescents to know these rights early and how they can exercise them responsibly. A community leader reported that: *“The SRH and rights should be taught in our schools so that adolescents can have clear understanding and exercise them with some responsibilities.”*(Key informant 11). Another community leader reported that: *“I think it is right time now that SRH and rights for adolescents should be taught in our schools for early preparation of our children*.” (FGD 4, respondent 12).

## Discussion

Taken together, the results of this study reveals the important roles performed by community leaders in addressing adolescent’s HIV and promote SRH and Rights in Mulanje, Malawi. Participants agreed that they have various roles towards promotion of the adolescents’ health.

### The advisory role on health life for adolescents

According to the study findings, community leaders are taking part in advisory role on adolescent’s health life. They do this by advising adolescents on the importance of abstinence and the need for HIV testing. In addition, community leaders indicated that they are encouraging those adolescents on ART to live positively and continue taking their drugs. This finding is similar with what was reported in another study in Zimbabwe by Dodo in 2013, where it was reported that traditional leaders were considered as mediators, judges and advisors whose verdicts were respected and taken with high esteem [19]. In another study conducted in Mulanje and Thyolo by Liwewe *et-a*l, it was reported that initiation counsellors were giving conflicting messages to the adolescents. The study further revealed that while initiation counsellors advised adolescents on the negative effects of the sexual intercourse, they were at the same time encouraging it [20]. This is in agreement with what our study has found as there were mixed feelings among the community leaders about advising adolescents on the SRH issues. This has also been reported in another study done in Mulanje, whereby, many of initiation counsellors claimed not to encourage girls to practice sex intercourse but at the same time they reported knowing others who do that [21]. This mixed information from community leaders mislead some of the adolescents who indulges in unsafe sexual activities thereby contracting HIV and early pregnancies which negatively affect their health. Therefore, our study ascertain the claims made by other studies on adolescents when it comes to addressing the SRH and HIV issues. Probably, this mixed information could be due to literacy level of the community leaders, or differences in religious backgrounds. We suggest civic education to all the community leaders on adolescent’s HIV, SRH and rights so that they should give correct information that can assist in reducing spread of HIV and promote SRH and rights.. We hope that the community leaders as advisors can help to improve the health of the adolescents by giving them information that is consistency.

### The encouragement role on safe behaviour towards prevention of HIV and SRH

Safe behaviour can assist in reducing HIV transmission and promote SRH practices among the adolescents. Our findings indicate that the community leaders are encouraging adolescents on safer behaviours towards prevention of HIV and promote SRH. The safer behaviours include VMMC and the cervical cancer prevention among adolescent boys and girls respectively. Although different study design approach was used but our findings are similar with what was reported in another study by Marashe J in Zimbabwe, where it was reported that traditional leaders were encouraging behavioral change among the youth and adults inorder to curb the spread of HIV. They further reported that the HIV pandemic could possibly be contained if government fully empowered the community leaders [22]. This study in Zimbabwe used mixed study design approach both qualitative and quantitative unlike our study. To concur with this, another study on perceptions of VMMC among circumcised and non-circumcised communities in Malawi [23] reported that community leaders especially those from religious background were encouraging VMMC among adolescents. Although our findings are similar with other studies which suggest that adolescents are given more information to reduce HIV prevalence and early pregnancies, however this is not the case in Mulanje, Malawi. According to the District Health Management Information System (DHMIS) in Mulanje, it reported a 7.8% prevalence of HIV by 2018 amongst the adolescents. On the other hand, MDHS 2015–16 also reported a 31.6% prevalence of early pregnancy cases among adolescents in the district. This may suggest that, the provision of conflicting messages by community leaders can be among the possible contributing factors. The community leaders should give similar information to the adolescents if the fight against HIV is to be won and have an HIV free generation by the year 2030 as desired in Sustainable Development Goals.

### The role of regulating and restricting cultural practices and beliefs that fuel HIV and deterred SRH rights

Community leaders are highly regarded and respected within their communities. They can assist in the prevention of HIV among adolescents. It is reported that the village chief, who are community leaders, plays the principal role in establishing and maintaining norms as well as decision-making around communities [24]. The Malawi National HIV prevention Strategy 2015–2020 [25] included the need to address cultural practices and beliefs inorder to reduce the spread of HIV. Addressing cultural practices and beliefs which fuel HIV transmission can also assist to promote SRH. The results of this study indicate that the community leaders regulate and restrict harmful cultural practices and beliefs that can fuel the spread of HIV and deterred SRH within their communities. There are number of cultural practices in Malawi. In Mulanje district, for example, one of the prominent cultural practices is the initiation ceremonies championed by the community leaders. This study reported that the community leaders restricted local male circumcision performed during initiation ceremonies. Our findings on restricting cultural practices are similar with what was reported by the UNFPA that one of the female chiefs from Malawi used her power to end harmful practices against girls and women by banishing gulewamkulu (masked men who perform ritual dances at traditional ceremonies) who was harassing women [26]. It is imperative that the community leaders are taking part in encouraging changes, which calls for a different approach to the longstanding traditional practices that are harmful to the adolescents.

To this far, we expected reduction in HIV infection and early pregnancies among adolescents in Malawi and Mulanje district in particular which is not the case. This may suggest that majority of the adolescents in Mulanje are not taking the restrictions on addressing harmful cultural practices seriously due to the inconsistency of information provided by the community leaders. This has negative impact on the lives of adolescents as the spread of HIV is increasing and more adolescents are getting early and unplanned pregnancies. Therefore, changing harmful traditional practices is a complex process that must involve all stakeholders including the community leaders and their subjects. This can be achieved if parents and the community leaders could have common goals in addressing SRH issues affecting adolescents.

### The role of formulating and advocating for bye-laws that guide adolescent SRH

The majority of community leaders reported that they formulate and advocate bye-laws within their communities to ensure that adolescents are protected from forced or coerced sexual intercourse and early marriages. Though not applicable national wide, however, bye-laws have shown to be an important tool that can help in health promotion and uplifting lives of adolescents. Our finding is similar with what was reported in another study conducted in Mangochi, Malawi, by Mamba K.C *et-al*. They reported that bye-laws were among reasons that forced pregnant women to start antenatal clinics early as they were afraid to be fined, which is similar with what we have found [27]. Although most of these bye-laws are formulated to address specific problem, for example maternal deaths, we feel that they can also be applied towards improvement of adolescents’ health.

However, another study reviewed indicated that, some bye-laws formulated by the community leaders limit access to health services. A qualitative study done in Ntcheu district, Malawi, on understanding barriers preventing pregnant women from starting antenatal clinic in the first trimester of pregnancy by Chimatiro S.C *et-al,* reported that, absence of male partners prevented women from starting antenatal clinic early, following the introduction of bye-laws that every women should be accompanied by her partner [28]. This implies that, the role of formulating and advocating for the bye-laws by community leaders is very essential much as it brings an improvement to the lives of their subjects without restricting access to health services. Moreover, only bye-laws that can promote health services uptake among the adolescents should be championed without restrictions. We presume that, Mulanje as a district and Malawi at large could do better in addressing adolescent’s HIV and improve SHR and rights if the formulated bye-laws are user friendly, free of restrictions.

### Dealing with the sexual abuses

Domestic violence is common in Malawi as it has been reported that 34% of women experienced it since their adolescents age [29]. Sexual abuse is one of the common domestic violence experienced by most adolescents in Malawi including Mulanje district [30]. The findings of this study indicated that community leaders are taking part in dealing with the sexual abuse among adolescents. This finding is in line with what was reported in South Africa, where it indicated that traditional leaders were addressing cultural practices that predisposes communities to HIV and gender-based violence [31]. Although this study done in South Africa did not specifically reported on the sexual abuse among adolescents, however, it is worth noting that community leaders are on the forefront addressing gender based violence which include sexual abuses.

The community leaders, however, highlighted that they depend on the adolescents to report and disclose information relating to the abuse she/he has been subjected to. This means that community leaders are limited on addressing sexual abuse as some of the adolescents do not report such conducts. This is in line with what was reported during national survey on violence against the children and young women in Malawi that most of the adolescents are less likely to report sexual abuse especially one done by their relations [30]. Moreover, it is indicated that the traditional leaders are major providers of justice [32] implying that they can assist in ending domestic violence including sexual abuse. In an evaluation of the joint programme on adolescent girls in Malawi, it was reported that girls were advised to report cases of abuse to appropriate authorities and structures including community leaders such as village headmen [33]. Further, the involvement of community leaders in dealing with domestic violence including sexual abuse is very important in the promotion of adolescents’ health. The results of evaluation of the project on the “Traditional Leaders Championing Prevention of Domestic Violence in their Communities Project in Lesotho and Malawi,” indicated that 9 in every 10 traditional leaders reported a decline in prevalence of domestic violence after their involvement [34]. This study, suggests that community leaders can assist in bringing much needed change in dealing with sexual abuse to improve adolescents SRH and reduce spread of HIV. Therefore, we suggest that adolescents should be sensitized to report any form of abuse they face in their day to day activities. This may help the community leaders to ably act on the reported abuses on time.

### Promotion of the youth friendly health services

YFRHS are not well known among majority of the community leaders as this current study indicates. It has been reported somewhere that YFRHS awareness and use is low especially in rural areas which is consistence with our finding [33]. Moreover, it was also reported that culture was one of the major factors that prevented most adolescents from accessing the YFRHS. Furthermore, the study reported that there is high resistance from the religious and community leaders to discuss sexual issues with adolescents [35]. The poor knowledge and low usage of the YFRHS among many of adolescents in Malawi was also reported by Zombe [36]. Although these studies used different study designs and methodologies, however, the findings are similar. The studies indicated that the YFRHS works so well in addressing issues of adolescents’ health. However, our study has reported that there is limited knowledge among the community leaders on the YFRHS. We, therefore, suggest that this could be one of the associated factors limiting accessibility of SRH services among the adolescents. Further, we suggest continuous awareness and engagement of the community leaders on YFRHS which can help to improve SRH services accessibility among adolescents. The community leaders and elders should feel free to discuss HIV and SRH issues including sexual activities with adolescents. By openly discussing sexual activity issues, adolescents can have time to learn which can lead to the change of their mind set. This can assist to reduce HIV prevalence and improve SRH among most of the adolescents.

## Conclusion

Our study suggests that the roles of community leaders differs depending on the position held and institution represented. Those community leaders from the religious back ground have shown to have different views with those community leaders representing other institutions not affiliated to religion. However, our study has shown that there is general agreement among all community leaders on the challenges faced as far as adolescent HIV and SRH issues are concerned in Mulanje, Malawi. In addition, the study has also shown that community leaders, irrespective of their positions and institutions represented are playing various roles as far as adolescents HIV and SRH and rights, are concerned. However, this current study noted that the community leaders are providing conflict ideas as they perform these duties. The conflicting information given by community leaders may potentially be the leading cause for the failure to thrive of HIV and SRH services in the society amongst majority of the adolescents. Based on this, we recommend continuous community engagement so that community leaders should perform their roles without conflicting one another. Importantly, community leaders should be organized and come together so that they can learn from one another on how they can approach adolescents with one unique voice inorder to reduce further spread of HIV and improve SRH and rights in this age group.

We have further indicated that there is limited knowledge among community leaders on adolescent SRH rights including YFRHS. Therefore, we suggest continuous community awareness and sensitisation in Mulanje district, Malawi, inorder to equip community leaders with adequate knowledge about SRH rights for the adolescents. Continuous community sensitisation and awareness will provide a learning chance for the community leaders to have more information on YFRHS. This, in turn, will assist community leaders to encourage adolescents on YFRHS resulting into improved service accessibility. Furthermore, we recommend that stakeholders involved in the fight against HIV and SRH rights should invest more on capacity building among the community leaders.

## Data Availability

Data for this study can be accessed at University of Malawi College of Medicine Library and from the corresponding author upon request.

## Abbreviations

HAS: Health Surveillance Assistant
HIV: Human Immunodeficient Virus
SRH: Sexual and Reproductive Health
MDHS: Malawi Demographics and Health Survey
YFRHS: Youth Friendly and Reproductive Health Services
